# Prolonged indwelling catheter time after RARP does not lead to follow-up surgery

**DOI:** 10.1007/s00345-024-05080-4

**Published:** 2024-06-18

**Authors:** Sebastian Lenart, Markus Holub, Georg Gutjahr, Ingrid Berger, Anton Ponholzer

**Affiliations:** 1 Department of Urology and Andrology, St. John of God Hospital Vienna – Krankenhaus der Barmherzigen Brüder Wien, Johannes-von-Gott Platz 1, Vienna, 1020 Austria; 2 Department of Mathematics, Center of Research in Analytics and Technologies for Education, Amrita Vishwa Vidyapeetham, Amritapuri, Kollam India

**Keywords:** RARP, Prolonged catheterization, Complications after RARP

## Abstract

**Background:**

Indwelling catheterization following radical prostatectomy is used to aid healing and urinary drainage. While early removal is well investigated, prolonged catheterization has only been investigated in terms of urinary incontinence. Other complications such as anastomotic strictures are unexplored so far. This study aims to analyze the sequelae of catheterization lasting more than 14 days after robotic-assisted radical prostatectomy (RARP).

**Methods:**

A prospective database of 3087 patients undergoing RARP was analyzed, focusing on 180 patients with catheterization exceeding 14 days (Group A) and 88 matched controls (Group B). Outcome measures included subsequent surgeries, complications, and functional outcomes.

**Results:**

Prolonged catheterization did not significantly increase the need for subsequent surgeries (6% in Group A vs. 7% in Group B, *p* = .95). However, anastomotic strictures were more common in Group A (3%) compared to Group B (0%) after exclusion of risk factors. Incontinence rates were similar between groups, although a subgroup analysis revealed higher incontinence rates in patients with catheterization exceeding 28 days. No significant differences were observed in erectile function or quality of life between the groups.

**Conclusion:**

Prolonged catheterization after RARP does not independently increase the risk of anastomotic strictures in the general population. However, in patients without risk factors, prolonged catheter dwell time may elevate the risk of strictures and subsequent surgeries. Additionally, patients with catheterization exceeding 28 days may experience higher rates of long-term incontinence. Further studies with larger sample sizes are needed to confirm these findings and elucidate the long-term implications of prolonged catheterization.

## Introduction

The ratio for indwelling catheter after radical prostatectomy is to support healing of the anastomosis and allow urinary drainage. It is usually kept for 4–10 days, but several studies showed that anastomosis is watertight already immediately after surgery, explaining uncomplicated courses even after removal on the 2nd day after surgery [1, 2]. While early removal can avoid complications like urinary tract infections and improve quality of life, an increased risk for acute urinary retention due to tissue swelling could be observed [3, 4]. Multiple studies investigated and discussed the possibility of early catheter removal, but only a few analysed sequels due to prolonged catheterization [5, 6]. Whereas worse short- and intermediate urinary continence has been shown, long-term continence seems to be similar in comparison to short catheter time [7]. Other complications, like the development of strictures and especially the need for further surgery as a result of prolonged catheterization, have not been investigated so far. The aim of this study is to analyse the sequel of prolonged catheterization of more than 14 days after robotic-assisted radical prostatectomy (RARP).

## Material and methods

### Patient population

We analysed a prospectively maintained database with 3087 patients starting in 2011 for patients with prolonged catheterization of more than 14 days due to insufficient anastomosis after RARP. Anastomosis was assessed with a standardized retrograde instillation of 100 ml 50% cystografin during X-Ray fluoroscopy. Our standard operating procedure intended cystography between the 4th and 7th day after surgery, depending on surgeon’s preference. In cases of any leakage, the indwelling catheter remained for another week, and another assessment was performed. We included all patients with a catheterization time of longer than two weeks with leakage in the routinely performed first cystogram and leakage on the 14th day after surgery. After excluding patients because of adjuvant radiation or prior TURP, 180 remained and were considered for further analysis (Group A). We introduced a matched pair control group 2:1 with catheterization less than 7 days with matching criteria: age, BMI, prostate weight, PSA value, and surgery time (Group B, *n* = 88) in order to exclude objective influencing parameters. Group A and B patients filled out an adapted version of the EPIC questionnaire and additional questions concerning surgeries after RARP [8]. All pre- and postoperative data were collected routinely in our institutional database, with all patients agreeing on data collection by informed consent. All surgeons were highly experienced robotic surgeons with 50 RARP per year in average.

### Statistical analysis

The primary endpoint was defined as any surgery after RARP. Secondary endpoints were infectious complications like symphysitis, potency, and incontinence. Symphysitis was defined as clinical and imaging inflammation in the area of the symphysis, requiring antibiotic treatment.

Quantitative variables were presented by means and standard deviations. Categorical Nominal variables were described as percentages. To address the potential confounding effect resulting from unbalanced response patterns to the surveys, the analysis employed the inverse probability of treatment weighting (IPTW) method. The propensity score was calculated from the baseline characteristics of age, BMI, prostate weight, pT, and operation duration. Propensity scores were calculated for the comparison of 14 and 28 days. For comparing outcomes between the groups (either less than 14 versus at least 14 days, or less than 28 versus at least 28 days), Poisson regression was used for count data, logistic regression for binary outcomes, and least squares regression for scores. A non-inferiority test with a preserved fraction of 50% was applied for the primary outcome.

## Results

### Clinical aspects

Out of the 268 patients (Group A and B) contacted, we received questionnaires back from 132 patients (49,3%, A: 89; B: 43). In median, indwelling catheter time in A was 28,94 (SD 10,62) and 5,7 days in B (SD 1,55). 

Table 1 shows baseline characteristics and the difference between the two groups, either unadjusted or using IPTW. After IPTW, no significant differences between baseline characteristics remain.

**Table 1:** baseline characteristics of patients stratified by catheter duration groups

	Group A		Group B		Difference (unadjusted)		Difference (IPTW)		*p*-value for difference
	Mean	SD	Mean	SD	Mean	SD	Mean	SD	
Age	67,4	6,5	61,5	7,3	5,9	1,3	0,9	1,3	0,45
BMI	28,3	4,4	26,3	3,8	2,0	0,8	0,7	0,7	0,32
Prostate weight	57,1	20,2	48,9	11,9	8,2	3,3	4,2	2,8	0,13
pT	3,2	1,0	3,1	1,1	0,1	0,2	-0,1	0,2	0,71
Surgery duration	158,9	48,6	145,1	42,4	13,8	8,7	1,4	8,0	0,87

The histological result was not a parameter for stratification. However, the distribution of Gleason scores differed significantly between A: ISUP 1 = 14 (15.7%), ISUP 2 = 33 (37.0%), ISUP 3 = 25 (28%), ISUP 4 = 8 (9%), ISUP 5 = 9 (10.2%) and B: ISUP 1 = 12 (27.9%), ISUP 2 = 33 (37.1%), ISUP 3 = 7 (16.3%), ISUP 4 = 5 (11.6%), ISUP 5 = 0; LR-Test: *p* = .02

### Complications and surgery due to prolonged catheterization

Of all patients, 13 (9.8%) experienced any complication in causal relation to RARP, 8 (9%) cases in Group A and 5 (11.6%) cases in Group B (OR = 1.3; 95% CI [0.5–3.3], *p* = .55). Clavien Dindo complication ≥ III were described in 9 cases (A: 5; B: 4), 8 of which were anastomotic stricture incision, one was a lymphocele drain. A necessity for surgery in relation to RARP was observed in 5 (6%) cases in A and 3 (7%) cases in B (OR = 1.0; 95% CI [0.4–2.7], *p* = .95). T-test did not show significant differences between the incidence of anastomotic strictures and duration of indwelling catheter (mean difference: 1.19; *p* = .86) (see Table 2). Furthermore, a non-inferiority test with a preserved fraction of 50% for the primary outcome, “Have you undergone any surgery related to prostatectomy?” was highly significant (*p* < .01). This means that patients with prolonged catheterization did not have a high risk of requiring surgery if baseline characteristics were matched. Despite differences between Gleason Score distribution and incidence of prolonged catheterization, no impact on primary or secondary endpoints were measurable.

**Table 2:** Incidence of necessity for any further treatment, complications, and surgery related to prostatectomy

		Group A	Group B		*p*
Any treatment	no	79	39	118	0,73
	yes	10	4	14	
Surgery	no	81	38	119	0,95
	yes	5*	3	8*	
Complications	Epididymitis	1	0	1	0,55
	Anastomosis stricture*	5*	3	8*	
	Lymphocele	0	1	1	
	Macrohaematuria	0	1	1	
	Symphysitis*	2*	0	2*	
		89	43	132	

*1 patient in Group A experienced concomitant symphysitis and anastomosis stricture

### Functional results after RARP due to prolonged catheterization

Adapted EPIC-26 Continence data were available in 96,97% (*n* = 128) of the patients (A: 64.4%, *n* = 85; B: 32.6%, *n* = 43). Continence rates were primarily measured based on the number of pads used. According to this criterion, 52.9% (*n* = 45) patients from A and 62.8% (*n* = 27) from B were considered continent. Required pad use between A and B distributed as follows: one pad: 32.9% (*n* = 28) vs. 30.2% (*n* = 13); two pads: 4.7% (*n* = 4) vs. 2.3% (*n* = 1); three pads: 9.4% (*n* = 8) vs. 4.7% (*n* = 2); OR = 1.1; 95% CI [0.8–1.5]; *p* = .52). We analysed a subgroup of patients with super long-term catheterization since median duration time was 28 days (49%, *n* = 44). In this subgroup analysis with a catheterization duration of over 28 days, incontinence was observed in 80% of cases (one pad: 33.3%, *n* = 2; three pads: 33.3%, *n* = 2; OR = 1.4; 95% CI [1.9–1.9]; *p* = .03) (see Table 3).

Differences for the use of medication to maintain erectile function could be identified (OR = 0.8; 95% CI [0.7–1.0]; *p* = .01), but not for the group of super long-term catheterization. Besides, we did not observe any differences in erectile function and quality of life between groups A and B, nor the subgroup of patients with super long-term catheterization.

Only the question asking for pad count and residual urine volume have shown significant differences between the two groups.

**Table 3:** Comparison of clinical outcomes between the two groups using IPTW to balance baseline characteristics

		Group A vs. Group B				Group A ≥ 28 days			
type	variable	Effect	95% CI		p	Effect	95% CI		p
count	number of pads	1,1	0,8	1,5	0,52	1,4	1,0	1,9	0,03
binary	Did you take psychological support in the last 4 weeks?	3,6	0,9	15,0	0,08	1,0	0,4	2,7	0,97
	Have you undergone any surgery related to the prostatectomy?	1,0	0,4	2,7	0,95	0,7	0,3	1,8	0,49
	Did you have any kind of complications related to the prostatectomy?	1,3	0,5	3,3	0,55	1,2	0,5	2,7	0,64
	Did you receive hormone therapy after the prostatectomy?	1,1	0,6	2,3	0,72	0,8	0,4	1,6	0,52
	Did you receive any medication related to the prostatectomy?	0,4	0,2	1,1	0,07	0,8	0,3	2,5	0,75
score	How often did you experience urinary incontinence in the last 4 weeks?	1,0	0,6	1,9	0,87	1,1	0,6	1,9	0,86
	How has your urinary voiding control been in the last 4 weeks?	0,9	0,7	1,2	0,64	1,1	0,9	1,5	0,34
	How often has urinary incontinence been problematic for you in the last 4 weeks?	0,8	0,5	1,2	0,22	1,1	0,7	1,6	0,62
	How often has burning during urination been problematic for you in the last 4 weeks?	1,1	0,9	1,3	0,31	1,1	0,9	1,3	0,37
	How often have you noticed blood in your urine in the last 4 weeks?	1,1	0,9	1,3	0,27	1,0	0,8	1,2	0,97
	How often has incomplete bladder emptying been problematic for you in the last 4 weeks?	1,1	0,8	1,5	0,58	1,4	1,0	2,1	0,05
	How often has frequent daytime urination been problematic for you in the last 4 weeks?	0,8	0,5	1,1	0,16	1,4	1,0	2,1	0,09
	All in all, how severe have your urinary symptoms been in the last 4 weeks?	0,7	0,5	1,0	0,04	1,1	0,8	1,7	0,48
	How has your ability to have an erection been in the last 4 weeks?	0,7	0,5	1,1	0,16	0,9	0,6	1,4	0,78
	How has your ability to have an orgasm been in the last 4 weeks?	0,8	0,5	1,2	0,29	1,0	0,6	1,7	0,88
	How would you describe the quality of your erection in the last 4 weeks?	0,7	0,5	1,0	0,06	0,8	0,6	1,2	0,37
	How often have you had erections in the last 4 weeks?	0,7	0,4	1,1	0,12	0,9	0,6	1,5	0,69
	How would you describe your overall sexual function in the last 4 weeks?	0,8	0,5	1,2	0,25	1,0	0,7	1,5	0,97
	How much have sexual function issues or their absence been problematic for you in the last 4 weeks?	1,2	0,8	2,0	0,38	1,1	0,7	1,8	0,72
	Have you taken any potency-enhancing medications to maintain an erection in the last 4 weeks?	0,8	0,7	1,0	0,01	1,0	0,9	1,2	0,73
	Have you used any erection aids to achieve an erection in the last 4 weeks?	1,0	0,9	1,1	0,98	1,0	0,9	1,1	0,70
	How would you rate your quality of life?	0,5	0,2	1,2	0,12	0,4	0,2	1,0	0,06

## Discussion

Prolonged catheterization occurs in approximately 10–15% after radical prostatectomy [5]. It is necessary in case of intraoperative or postoperative anastomosis leakage. Usually, a conservative approach is preferred to surgical repair of the anastomosis, as prolonged catheterization duration sufficiently prevents urinoma, and the extravesical vesicourethral cavity forms epithelialization over time. While many studies have already investigated the ideal time of catheter removal or the earliest possible timing for catheter removal after RARP or even catheter removal without cystogram, fewer studies have examined the sequel of prolonged catheter dwell time due to insufficient anastomosis [9, 10]. Moreover, those studies primarily explored stress urinary incontinence post-RARP, since besides oncological outcomes, particularly urinary continence and erectile function, are considered the trifecta goals of RARP [7]. Neither the need for further surgery due to prolonged indwelling catheter, any kind of complications nor implication on functional results or life quality have been investigated so far.

The primary endpoint of this study was the rate of subsequent operations following RARP in association with prolonged catheterization duration. Here, all urogenital tract complications and surgeries were recorded. Overall complications occurred in 8 cases of Group A (9%) and 5 cases of Group B (11.6%). In A, 5 of those patients (6%) required a subsequent operation, while in B, 3 of those patients (7%) required surgery. While non-surgical complications included macrohematuria (Group < 14: 1, > 14: 0), epididymitis (< 14: 0; >14:1), and symphysitis (< 14:0; >14:2), the reason for required surgery was anastomotic strictures in all cases. Strictures were diagnosed using cystoscopy and treated with stenosis incision.

Anastomotic stenosis is a rare but known sequelae of prostatectomy. It has a reported incidence of 2.7–15% after open radical retropubic prostatectomy and 1–3% after RARP in the literature [11–14]. Several studies investigated the reasons and risk factors for developing anastomotic stricture. The known risk factors for anastomotic strictures, in general, include infections, diabetes mellitus, and previous prolonged catheterization. The strongest risk factor for stenosis after prostatectomy was identified as diabetes mellitus too, the number of sutures in the anastomosis, and the surgeon’s experience as well as adjuvant or salvage radiation for prostate cancer [12]. The catheterization duration in the context of prostatectomy has not been investigated as a risk factor in any studies so far.

In both of our groups, the incidence of anastomotic strictures was higher than reported in the literature. However, upon closer examination, it was found that in Group B, all patients with anastomotic strictures had a risk factor for stricture formation: one patient had had transurethral treatment for bladder cancer and developed a stricture three years after RARP and one year after bladder treatment. Another patient had a history of urethral stricture and developed a stricture two years after RARP, albeit at the anastomotic site. The third patient had a history of recurrent ureteral stones and underwent procedures with ureteroscopy, leading to more frequent catheterization. In Group A, none of the five patients with anastomotic strictures had any of these risk factors, and the stricture occurred on average one year after RARP (at 5 months, 6 months, 1 year, 2 years, 3 years). Thus, if patients with risk factors are excluded, anastomotic strictures only occurred in the group with prolonged catheterization. However, we did not observe differences in the incidence of anastomotic stricture between the prolonged indwelling time of more than 14 days (60%, *n* = 3, Md = 17.3 days) versus the superlong catheterization duration of more than 28 days (40%, *n* = 2, Md = 47.5 days). Therefore, prolonged catheterization cannot be considered independent risk factor.

Yet, all patients with symphysitis belonged to the subgroup with catheterization duration over 28 days (*n* = 2, Md = 33.3 days). However, due to the study methodology and the extremely low number of cases, no relevant conclusions can be drawn from this. Filipas et al. assessed complications in a cohort of patients with insufficient anastomosis and prolonged catheterization after open prostatectomy (ORP) and RARP and found anastomotic strictures (ORP 11.1%, RARP 2.3%) and symphysitis (ORP 0.0%, RARP 18.6%), but the timepoint of follow-up was heterogenous, thus possibly overlooking of events [15].

Our study met the secondary endpoint and could confirm the results of most of the other studies: prolonged catheter dwell time leads to lower early continence. In the study with the largest sample size to date, Tilki et al. describe poorer early and intermediate continence rates for the group with catheter duration > 15 days, while no difference could be observed after one year. Her work aligns with the findings in the literature: Palisaar et al. found similar results for the patient group with a prolonged catheterization time of 8–14 (OR = 1.4, *p* = .001) and ≥ 15 days (OR = 1.6, *p* < .001) compared to ≤ 7 days. This study also examined ORP with the same results [16]. Another study by Cormio et al. found poorer early continence after 3 months in patients with a catheter dwell time of even > 7 days (34.2 vs. 77.5%, *p* < .001) [17]. However, the studies are not directly comparable to each other because the definition of incontinence was different in each study.

Our study only examined long-term continence rates with follow-up assessment by questionnaires 5 years after surgery at least. Incontinence was simply defined as pad use per day. We did not find any difference between groups A and B (OR = 1.1; 95% CI [0.8–1.5]; *p* = .52), matching the results in literature. However, in a subgroup analysis involving patients with a catheter dwell time of > 28 days, poorer continence rates were indeed observed, as measured by the number of pads required per day (Group A: 47% ≥1, 33%: 1, 5%: 2, 9%: 3; Group B: 37% ≥1, 30%: 1, 2%: 2, 5%: 2; OR = 1.4; 95% CI [1.0–1.9]; *p* = .03). None of the studies in the literature made this distinction regarding “very long-term catheter users”. Except for the higher number of pads, we could not identify any other risks for the Group of indwelling catheter time 28 days though. Neither the rates of follow-up surgeries (LR = 3.65; *p* = .30) nor the rate of other complications (LR = 2.46; *p* = .48) were higher in this group.

However, our study is not without limitations: Due to its retrospective nature, addressing a research question is inevitably somewhat incomplete. Since we only processed data from patients from whom we also received a response, the population of those patients who had a prolonged indwelling catheter time was not completely homogeneous. We identified patients from the database and then contacted them. The response rate was 49.6%. Therefore, a certain selection bias cannot be ruled out. Although we were able to identify associations and risks for anastomotic strictures as well as incontinence in the group of patients with super-long catheters, due to the low number of cases, no clear conclusions can be drawn.

## Conclusion

An extended duration of indwelling catheterization due to a leaky anastomosis with an extravesical formation does not increase the risk of anastomotic strictures in a population-based cohort. However, in patients without risk factors for developing strictures, prolonged catheter dwell time may indeed increase the risk, necessitating follow-up surgeries such as stricture incision. Patients with catheter time 28 days had higher need for pads. Nonetheless, due to the small sample size, the results of this study do not allow for adequate conclusions.
